# The contribution of changes to tax and social security to stalled life expectancy trends in Scotland: a modelling study

**DOI:** 10.1136/jech-2020-214770

**Published:** 2020-10-20

**Authors:** Elizabeth Richardson, Martin Taulbut, Mark Robinson, Andrew Pulford, Gerry McCartney

**Affiliations:** 1 Place and Wellbeing Directorate, Public Health Scotland, Glasgow, UK; 2 The University of Queensland, Saint Lucia, Australia

**Keywords:** Health inequalities, Poverty, Public health policy, Social epidemiology, Socio-economic

## Abstract

**Background:**

Life expectancy (LE) improvements have stalled, and UK tax and welfare ‘reforms’ have been proposed as a cause. We estimated the effects of tax and welfare reforms from 2010/2011 to 2021/2022 on LE and inequalities in LE in Scotland.

**Methods:**

We applied a published estimate of the cumulative income impact of the reforms to the households within Scottish Index of Multiple Deprivation (SIMD) quintiles. We estimated the impact on LE by applying a rate ratio for the impact of income on mortality rates (by age group, sex and SIMD quintile) and calculating the difference between inflation-only changes in benefits and the reforms.

**Results:**

We estimated that changes to household income resulting from the reforms would result in an additional 1041 (+3.7%) female deaths and 1013 (+3.8%) male deaths. These deaths represent an estimated reduction of female LE from 81.6 years to 81.2 years (−20 weeks), and male LE from 77.6 years to 77.2 years (−23 weeks). Cuts to benefits and tax credits were modelled to have the most detrimental impact on LE, and these were estimated to be most severe in the most deprived areas. The modelled impact on inequalities in LE was widening of the gap between the most and least deprived 20% of areas by a further 21 weeks for females and 23 weeks for males.

**Interpretation:**

This study provides further evidence that austerity, in the form of cuts to social security benefits, is likely to be an important cause of stalled LE across the UK.

## INTRODUCTION

The upward trend in life expectancy (LE) across the UK nations stalled around 2012–2014.^1^ This stalling presents one the greatest challenges to population health since the 1940s,^2^ with the lost gains in LE being similar to the worst case scenario of pandemic COVID-19.^3 4^ While a slowdown in improving LE was observed across many high-income countries, it was not seen everywhere, with the UK nations and USA among the worst affected.^1^ Various causes for this stalling in the UK have been proposed, including the impact of the cuts and freezes to benefits paid to low-income families and children since 2010/2011. These changes were part of a wider package of reduced public spending and ‘welfare reform’, aimed at the reduction of public sector debt and the public sector deficit.^5 6^

Scotland was also exposed to these UK Government policies. By 2021, welfare reforms are anticipated to have resulted in cumulative losses of around £630 per year for every working-age adult in Scotland.^7^ However, this average figure will understate the financial impact on those affected, since losses will be concentrated on individuals and households claiming benefits and tax credits. The distributional effects of changes to taxes and benefits on households’ incomes are likely to be regressive.^8^ The Scottish Government, along with other devolved administrations, chose to mitigate against some of the UK welfare reforms (eg, the ‘Bedroom Tax’—a reduction in housing benefit where the house is deemed to be under-occupied; and the abolition of Council Tax Benefit and the Social Fund).^8^ Given that poverty and income inequality are important causes of health inequalities,^9^ understanding the cumulative impact of real-world policy decisions that change households’ incomes could help inform policy-makers’ choices.

In this paper, we estimate the effects of tax and welfare reforms between 2010/2011 and 2021/2022 on LE and inequalities in LE in Scotland (in the absence of the additional anticipated impacts of COVID-19). In order to estimate the potential impact of these policy decisions on health, we used the Informing Interventions to reduce health Inequalities (‘Triple I’) scenario modelling approach. This approach was previously used to model the health impacts of a range of other income-based policies, as well as changes in employment.^10–12^

## METHODS

### General overview

We used published data on the cumulative effects of 2010/2011 to 2021/2022 fiscal policies (implemented or planned to be implemented) on household incomes,^8^ and used the Triple I scenario modelling tool to estimate how these household income changes would affect LE.

### Effects of the changes on household income

The modelling takes into account all changes to tax and social security policy made or planned for the period between May 2010 and 2021/2022, as well as the increases in the national minimum wage for employees aged 25 years and over (box 1).

**Box 1 Changes to taxes and benefits modelled by Portes and Reed (2019)^8^**

- Income Tax
- National Insurance Contributions (NICs)
- Indirect taxes (Value Added Tax and excise duties)
- Means-tested and non-means-tested social security benefits
- The benefits cap
- Tax credits
- Universal Credit (UC)
- Real-terms increases in statutory minimum wages including the National Living Wage (NLW) for employees aged 25 years and over.

The Portes and Reed report provides full details of the data sources and modelling methodology.^8^ Briefly, a micro-simulation model was used to estimate the impacts of implemented or planned tax and welfare reforms. The impacts on net household incomes (before housing costs) were calculated relative to a baseline scenario in which benefit and tax credit levels changed only in line with inflation each year. The authors provided us with the changes in household incomes for each type of tax/welfare reform and of selected specific policies (table 1), by income decile. We created quintiles by calculating the mean income for decile pairs (online supplemental appendix 1).

**Table 1:** Estimated percentage change of tax and welfare reforms (from 2010/2011 to 2021/2022) on equivalised net household income (before housing costs) by SIMD 2016 quintile

	SIMD 2016 quintile				
	1 (most deprived)	2	3	4	5 (least deprived)
**Tax/welfare reform**					
Benefits and tax credits*	−6.43	−5.42	−4.82	−4.04	−3.10
Universal Credit (UC)†	−0.16	−0.18	−0.29	−0.24	−0.20
Income Tax and NICs	1.58	1.52	1.51	1.35	1.20
Gross incomes‡	2.05	1.82	1.72	1.50	1.25
Indirect taxes	−1.47	−1.35	−1.28	−1.16	−1.04
All tax/welfare reforms	−4.43	−3.60	−3.16	−2.59	−1.88
**Specific policy reforms**					
DLA-PIP transfer§	−0.14	−0.14	−0.13	−0.12	−0.10
Post-2015 freeze¶	−0.81	−0.66	−0.57	−0.47	−0.35
Two child limit**	−0.35	−0.28	−0.24	−0.19	−0.13
UC work allowances††	−0.57	−0.47	−0.40	−0.33	−0.24
Scotland-specific reforms††	−0.29	−0.37	−0.41	−0.51	−0.62

*The impacts of all reforms to benefits and tax credits with the exception of Universal Credit.

†The additional impact of Universal Credit on incomes, after all other reforms to benefits and tax credits.

‡Changes to gross incomes as a result of real-terms increases in statutory minimum wages.

§The replacement of Disability Living Allowance (DLA) with Personal Independence Payment (PIP).

¶The four-year freeze in uprating of benefits, tax credits and Universal Credit for working-age individuals and families from 2016/2017 to 2020/2021.

**The two-child limit on Housing Benefit, tax credits and Universal Credit for children born after April 2017.

††Reductions in the tax-free work allowances for Universal Credit.

‡‡Scotland-specific reforms: making income tax slightly more progressive, a new Best Start Grant, and an increase to Carer’s Allowance.

NIC, National Insurance Contributions.

10.1136/jech-2020-214770.supp1 Supplementary data

To model the health effects of the changes using the Triple I modelling tool, we first required estimates of the effects of the tax and benefits changes on household incomes by area deprivation quintile (Scottish Index of Multiple Deprivation; SIMD 2016) rather than income quintile. To achieve that, we calculated the distribution of Scottish respondents to the 2014/2015 Family Resources Survey (FRS), by income quintile, between SIMD 2016 quintiles (online supplemental appendix 2). For example, our analysis estimated that 30% of the households in SIMD quintile 1 (most deprived) were in household income quintile 1 (poorest). The income quintile effects were weighted accordingly to give SIMD quintile estimates (eg, income change percentage for income quintile 1 would contribute 30% of the overall income change for SIMD quintile 1).

### The income-mortality relationship

The percentage changes in household income for the tax/welfare reform types and policies were used to estimate impacts on all-cause mortality risk for each SIMD quintile. The evidence base for the impact of income change on mortality has not yet been systematically synthesised (although authors on this paper are currently finalising such a review), although there is evidence from a number of robust studies that show increased income improves general health and mortality outcomes.^13 14^ We estimated the log-linear relationship between equivalised household income before housing costs (from the FRS 2014/2015, uprated to 2016 and linked to SIMD quintiles) and mortality rates for 2016 (derived from National Records of Scotland (NRS) data) (online supplemental appendix 3). The analysis estimated a mortality rate ratio of 0.454 per doubling of income. We applied this rate ratio to the income changes estimated for each SIMD quintile to predict impacts on mortality rates for that quintile (online supplemental appendix 4).

### Impacts of the changes on deaths

The effects of the tax and welfare reforms on the number of deaths were estimated by combining the mortality rate ratios with Public Health Scotland’s Triple I modelling approach. Triple I is described in detail elsewhere,^10 11 15^ but briefly, it is a tool for modelling the potential population health impact of various policies and interventions, using the best available effectiveness evidence. It estimates policy effects on a closed cohort: we used the Scottish adult population in 2016, grouped by sex, 5-year age group and SIMD 2016 quintile (NRS data). In the baseline scenario (population demographics as in 2016), we applied all-cause mortality rates (2016–2018; NRS data) to the population. The effects of each of the tax/welfare reform types and policies on all-cause deaths were modelled as separate scenarios. This was done by using the predicted effect of each on the mortality rate to adjust the baseline mortality rate.

Due to the aggregate 2010/2011 to 2021/2022 income change data we obtained, it was necessary to model effects on health as if this income change had been experienced in a single year. We made the pragmatic decision to use 2016, the middle year of the time period, such that some policies would be modelled before they occurred, but others would be modelled later. As the population would already have been exposed to some years of austerity policies by 2016, we assessed the impact of this decision on the results in a sensitivity analysis (see below).

### Impacts of the changes on life expectancy

We extracted numbers of deaths (by age group, sex and SIMD quintile) for the baseline and change scenarios from the Triple I modelling tool, and used these to estimate period LE at birth using the mean age at death from life tables. We then calculated the differences between the scenarios to estimate the change in LE that could be attributed to the changes in income due to the policy changes described in box 1.

### Sensitivity analyses

The greatest uncertainties in our models relate to the assumptions in the effect sizes rather than due to any sampling issues. We therefore tested the sensitivity of the results to the strength of the relationship between income and mortality by reducing the effect size by 25% and 50% (mortality rate ratios of 0.590 and 0.727, respectively).

We also assessed the sensitivity of the results to the choice of baseline year. We used population counts and mortality rates from 2010 (by age group, sex and SIMD 2012), and estimated the effects on mortality and LE of applying the cumulative income changes to this population.

As a third sensitivity analysis, we assessed the extent to which the estimated changes in LE due to income changes may have been offset by the potential positive population health impacts of increased employment over the same period. To do this, we used the Triple I employment model; full details are provided in online supplemental appendix 5.

## RESULTS

Due to the range of income levels represented within areas grouped by SIMD quintile, the impacts of the fiscal changes on household incomes varied less between SIMD quintiles (table 1) than between the original income quintiles (online supplemental appendix 1). We estimated that the combined effect of all tax and welfare reforms would reduce household incomes in all SIMD quintiles, but that those living in the most deprived areas would experience the biggest percentage decrease (−4.43%). This decrease was more than twice than that estimated for those living in the least deprived quintile (−1.88%). Changes to benefits and tax credits had the biggest detrimental impact on incomes (followed by indirect taxes and Universal Credit), and these were only partially mitigated for by increases in gross incomes resulting from reductions in Income Tax and National Insurance contributions (NIC).

Compared with baseline, we estimated that changes to household income resulting from 2010/2011 to 2021/2022 tax and welfare reforms would result in an additional 1041 (+3.7%) female deaths and 1013 (+3.8%) male deaths. LE at birth would decrease from 81.6 years to 81.2 years (−20 weeks) for females, and from 77.6 years to 77.2 years (−23 weeks) for males, all other things being equal. It should be noted that our baseline LE estimates are slightly higher than the official NRS estimates (81.1 years for females and 77.0 years for males for 2015–2017) because we used a different averaging period, modelled 5 year rather than single year age groups and did not include deaths for under 16 year olds (due to the design of the Triple I model).

**Figure 1:**
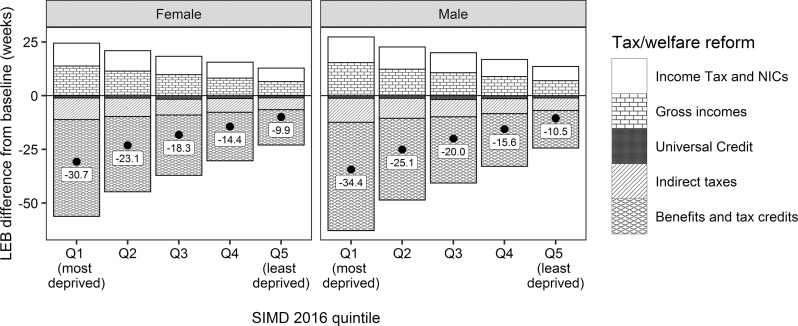
Estimated effects of tax and welfare reforms in Scotland (grouped by type) on life expectancy at birth (LEB) for (A) females and (B) males. The point estimate provided is the combined impact of all the tax and welfare changes.

Figure 1 shows the estimated impact of the tax and welfare reforms on LE, by SIMD 2016 quintile. The overall impact of the reforms on LE is estimated to be worst in the most deprived areas (−31 weeks for females and −34 weeks for males), and to have a smaller effect on those in the least deprived areas (−10 weeks for females and −11 weeks for males). It was estimated that cuts to benefits and tax credits would have the most detrimental impact on LE, and that these would be most severe in the most deprived SIMD quintile. Changes to indirect taxes and the introduction of Universal Credit would also have small detrimental impacts on LE across the population. Changes to gross incomes and Income Tax and NICs would result in increases to LE across the population, but these would be offset by the effects of the detrimental changes of reforms to benefits and tax credits (figure 1).

**Figure 2:**
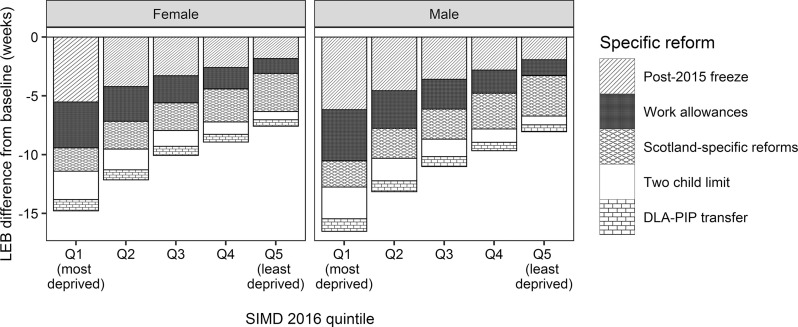
Estimated effects of specific tax and welfare reforms in Scotland on life expectancy at birth (LEB) for (A) females and (B) males.

Of the specific reforms modelled (figure 2), the post-2015 freeze on uprating of benefits and tax credits would have the biggest detrimental effect on LE in the most deprived areas (−6 weeks, both sexes), and the smallest effect on the least deprived areas (−2 weeks, both sexes). The Scotland-specific reforms (increasing Income Tax more for higher earners than for lower earners, a new Best Start Grant and an increase to Carer’s Allowance) would negatively affect the least deprived areas slightly more than the most deprived areas (−3 weeks compared to −2 weeks, both sexes).

In the baseline scenario (no changes to tax, inflationary changes only to welfare since 2010/2011) it was estimated that female LE in the least deprived areas was 7 years and 42 weeks higher than in the most deprived areas (10 years and 32 weeks for males). The household income changes resulting from all tax and welfare reforms between 2010/2011 and 2021/2022 were estimated to increase the gap by a further 21 weeks for females and 23 weeks for males.

### Sensitivity analyses

We first assessed the impact of attenuating the income and mortality relationship by 25% or 50%, and found that the effects on LE would be reduced by about one-third and one-half, respectively.

Moving the baseline year to 2010 resulted in small increases in the effect sizes of the reforms. All reforms combined were estimated to reduce LE at birth by 21 weeks for females and 24 weeks for males, compared with 20 and 23 weeks, respectively, in the main analysis (2016 baseline year).

We also checked whether increased employment over the period would offset the effects of the reforms, and found that the estimated increases to overall LE and reduced inequalities in LE associated with increased employment over the period would be much smaller than the detrimental effect of the tax and welfare reforms. As such, increased employment could not account for the scale of the estimated changes in LE and inequalities in LE.

## DISCUSSION

In this study, we have used scenario modelling to compare how changes to tax and welfare between 2010/2011 and 2021/2022 are likely to affect household incomes, deaths, LE and inequalities in LE in Scotland. Tax and welfare reforms were predicted to reduce LE by 20 weeks for women and 23 weeks for men over the period. Predicted reductions in LE were greatest in the most deprived areas—31 weeks for women and 34 weeks for men—and as a result the inequality between the least and most deprived fifths of the population widened by a further 21 weeks and 23 weeks for women and men, respectively.

An important strength of this study is that it models the contribution of specific interventions on health and health inequalities, which is likely to be valued by policy-makers.^16^ However, as with all modelling work, the findings should be interpreted in the context of the model specifications, assumptions and uncertainty. A key assumption was that the cross-sectional relationship between income and death rates would adequately predict the reduction in mortality that would result from an increase in income. While there is good evidence that increased income is likely to be causally related to improved health,^17^ the relationship could be weaker than our estimate, which would produce smaller effect sizes overall, as shown by the sensitivity analysis. Thus, the results of the income modelling presented here are likely to represent the upper limit of the potential effect. The effect sizes reported here may also be exaggerated by the assumption of Triple I that policy changes are applied simultaneously in year one, rather than being spread over 12 years, as they would be in the real world. However, we are interpreting the estimated change in LE as cumulative rather than as if it had occurred in a single year. Using 2016 as the baseline year was found to give slightly conservative effect estimates, compared to using 2010 (start of the study period). The greatest sources of uncertainty in our estimates are therefore due to the assumptions underlying the risk ratio used, rather than to any sampling issues. As such, we have not presented uncertainty intervals, as these could provide a false sense of confidence that the estimated results fell within a particular range.

The modelling here is limited to considering the direct income-related changes associated with tax and welfare reforms as a driver of health. It does not consider the many indirect drivers associated with welfare reforms which are likely to have undermined health, for example conditionality, benefit sanctions and new assessments for disability benefits.^18–20^ It also does not consider other policy changes likely to have improved health, for example the indirect positive impacts of participation in supportive employability programmes with a personal development component.^21^

In 2010, the UK government argued that the broader package of welfare reforms (including changes to taxes to make work pay and improved support to help people find work) would—by increasing employment rates and incomes from earnings directly and indirectly—be beneficial for health.^22^ An important question is therefore to what extent changes in employment associated with the welfare reforms modelled here will have offset any adverse impacts from income reductions seen through cuts and freezes to benefits. While we were unable to answer this directly, we were able to use the Triple I tool to explore to what extent overall changes in employment could compensate for any adverse health consequences associated with income loss (at a population level). This found that at the population level, the increase in LE from even substantial job gains would be more than cancelled out by the reductions associated with tax and welfare reforms (although it may not have been the same people impacted by both policies, online supplemental appendix 5).

Evaluating the impact of austerity policies in general, and the impact of changes to social security in particular, has been thus far limited to earlier time periods^23–25^ or self-reported health outcomes.^20 26 27^ Our study therefore helps to inform on the contribution that economic and social security policy may have made to the recent stalled LE trends, until definitive evaluation studies are available.^28^ Estimates of the extent to which LE has stalled are that by 2018 in Scotland (three-quarters of the way through the study period) LE was 56 and 60 weeks lower than predicted for women and men from trends from 1980, or 67 and 68 weeks lower than predicted for women and men from trends from 1990.^3^ We estimated LE reductions of 20 weeks for women and 23 weeks for men over the period, compared with a scenario without the austerity policies. If our estimates are accurate, the tax and welfare reforms could explain about one-third of the gap in LE between what has been observed and what was expected based on previous trends. Given this, the end of the benefits freeze and increase in the value of key working-age benefits, in April 2020, are welcome developments.

The study also provides knowledge relevant for decision-makers’ future choices on tax and spending, in the aftermath of the COVID-19 pandemic. In spring 2020, the UK Government announced a package of measures designed to mitigate the adverse economic impacts associated with the COVID-19 pandemic lockdown. The scale of the measures was large, and included increased spending on welfare as well as income-support measures for furloughed employees, the self-employed and small businesses. The Office for Budget Responsibility (OBR) anticipate that as a result of these measures, debt will rise to more than 90% of GDP and remain so until 2023–2024.^29^ Regressive changes to tax and spending since 2010/2011 were partly justified by the need to cut the debt quickly, because of a presumption that that level of public debt would stifle economic growth. While others have shown that this is unlikely to be the case,^30^ our findings suggest the cuts and freezes to benefits did have large adverse, unintended consequences for health and health inequalities. Our findings will allow policy-makers to take health into account when making decisions about how fiscal policy should respond to public debt as the economy recovers.

## CONCLUSION

This paper suggests that fewer people would have died, LE would have been substantially higher and health inequalities narrower, had the tax and benefit ‘reforms’ introduced since 2010/2011 (and planned until 2021/2022) not been implemented. Reversing the UK tax and welfare reforms since 2010/2011 and ensuring that the population have the incomes they require to live healthy and fulfiling lives should now be a public policy priority for the UK as we *build back better* from COVID-19.

**What is already known on this subject**

- Life expectancy improvements stalled in the UK around 2012–2014. Reductions in public spending are known to be associated with increased all-cause mortality and increased health inequalities. It is therefore possible that the UK’s austerity policies have contributed to the stalling of improvements in life expectancy.

**What this study adds**

- We estimate that the tax and benefit changes between 2010/2011 and 2021/2022 contribute to a reduction in life expectancy of 20 weeks for females and 23 weeks for males in Scotland, compared to the baseline scenario of inflation-only changes in benefits. Losses to life expectancy were greatest in the most deprived areas (31 weeks for females, 34 weeks for males), contributing to a widening in health inequalities. The health impacts of changes to tax and social security should be taken into account when considering how to allocate public finances, particularly in the aftermath of the COVID-19 outbreak.
