# Supplementary material for: The contribution of changes to tax and social security to stalled life expectancy trends in Scotland: a modelling study
Source: J Epidemiol Community Health. 2020 Oct 20;75(4):365–70. doi: 10.1136/jech-2020-214770 (PMC7958084; doi:10.1136/jech-2020-214770)
Supplement: Supplementary data [file jech-2020-214770supp001.pdf]

SUPPLEMENTARY INFORMATION

Supplementary Appendix 1

Percentage impact of tax and welfare reforms on equivalised household income (before housing costs), by household income quintile. 2021/22 tax year, Scotland. Derived from Portes and Reed (2019).[1]

	Household income quintile				
	1 (poorest)	2	3	4	5 (richest)
TAX/WELFARE REFORM					
Benefits and tax credits	-11·78	-6·44	-3·20	-1·57	-0·41
Universal Credit	1·86	-1·52	-0·95	-0·37	-0·05
Income Tax and NICs	1·19	1·93	1·95	1·67	0·39
Gross incomes	2·57	2·47	1·64	1·14	0·41
Indirect taxes	-2·15	-1·36	-1·24	-0·86	-0·63
All tax/welfare reforms	-8·31	-4·92	-1·80	0·01	-0·29
SPECIFIC POLICY REFORMS					
DLA-PIP transfer	-0·11	-0·16	-0·19	-0·14	-0·03
Post-2015 freeze	-1·71	-0·73	-0·28	-0·08	-0·01
2 child limit	-0·72	-0·41	-0·04	0·00	0·00
Work allowances	-1·13	-0·59	-0·19	-0·06	0·00
Scotland-specific reforms	-0·11	-0·19	-0·33	-0·59	-1·03

NIC: National Insurance Contributions

Supplementary Appendix 2

Percentage of the population of SIMD 2016 quintiles from each quintile of equivalised household income (before housing costs). Source: Weighted analysis of Scottish Family Resources Survey 2014/15 respondents[2] linked to SIMD 2016, conducted for Triple I.

	SIMD 2016 quintile				
Household income quintile	Q1	Q2	Q3	Q4	Q5
Q1	30·09	24·49	19·18	15·95	11·81
Q2	31·61	22·96	21·72	16·89	10·59
Q3	19·60	22·28	25·05	20·08	14·93
Q4	12·77	18·54	19·18	20·64	25·35
Q5	5·93	11·73	14·87	26·45	37·33
Total	100·00	100·00	100·00	100·00	100·00

__**Supplementary Appendix 3**__

Modelling the effects of income change on health

In the absence of evidence quantifying the effect of income change on mortality we regressed $\log_e$-transformed all-cause mortality rates (European age-standardised rates (EASR), calculated using data from National Records of Scotland[3] and the European Standard Population 2013[4]) on $\log_2$-transformed mean equivalised household income (before housing costs, values uprated to 2016 from FRS 2014/15[2]), for Scottish Index of Multiple Deprivation (SIMD) quintiles (see table below). The regression coefficient (β = -0·789 (standard error 0·036), p < 0·001, R^2 = 0·99) was exponentiated to give a mortality effect size of 0·454 per doubling of income.

Income and mortality data for SIMD quintiles, 2016.

SIMD 2016 quintile	Mean equivalised household income (£/month)	Mortality EASR per 100,000
Q1 (most deprived)	1,124·58	1,572
Q2	1,343·07	1,300
Q3	1,513·08	1,148
Q4	1,656·56	985
Q5 (least deprived)	1,967·32	843

Supplementary Appendix 4

Rate ratios for the estimated mortality effect of the tax and welfare reforms, by SIMD 2016 quintile.

	SIMD 2016 quintile				
	Q1 (most deprived)	Q2	Q3	Q4	Q5 (least deprived)
TAX/WELFARE REFORM					
Benefits and tax credits	1·079	1·065	1·058	1·048	1·037
Universal Credit	1·002	1·002	1·003	1·003	1·002
Income Tax and NICs	0·982	0·983	0·983	0·985	0·986
Gross incomes	0·977	0·980	0·981	0·983	0·986
Indirect taxes	1·017	1·016	1·015	1·013	1·012
All tax/welfare reforms	1·053	1·043	1·037	1·030	1·022
SPECIFIC POLICY REFORMS					
DLA-PIP transfer	1·002	1·002	1·002	1·001	1·001
Post-2015 freeze	1·009	1·008	1·007	1·005	1·004
2 child limit	1·004	1·003	1·003	1·002	1·002
Work allowances	1·007	1·005	1·005	1·004	1·003
Scotland-specific reforms	1·003	1·004	1·005	1·006	1·007

NIC: National Insurance Contributions

Supplementary Appendix 5

Do employment increases mitigate the effects?

We assessed to what extent increased employment over the period 2010/11 to 2021/22 – some of which may have been a result of the fiscal policies – would mitigate the estimated effects of the tax and welfare reforms. Annual Population Survey (APS) data for the total number of 16-64 year olds in employment in Scotland each year between 2010/11 to 2018/19 were extrapolated to give an estimate of 206,400 additional people moving into work between 2010/11 and 2021/22. We assumed that these new jobs would be distributed among the population as per the Scottish Government's breakdown of employment change between 2010 and 2017 by SIMD quintile (31%, 23%, 8%, 14%, and 25% from the most to the least deprived quintiles, respectively) (Scottish Government analysis of Annual Population Survey data, 2018). We used the Triple I model for job provision to estimate the effects of these additional jobs on numbers of deaths.

In the population as a whole, we estimated that these large-scale job gains over the period (n = 206,400) would result in fewer deaths (152 and 208 fewer for females and males, respectively), increased life expectancy, especially in the most deprived quintile, and narrower inequalities in life expectancy (see table). However, these positive outcomes would not be large enough to offset the detrimental effects of welfare reforms and other spending changes across the whole population. Moreover, the individuals whose incomes and health were adversely affected by the tax and welfare reforms may not be those that would have been positively affected by moving into employment. In other words, it is likely that at a population level the increase in life expectancy from substantial job gains would be more than cancelled out by the reductions associated with tax and welfare reform.

A limitation of this modelling is that we did not consider the quality of employment that those moving off long-term benefits are likely to secure, and note that not all jobs are good jobs. Mortality rates in elementary occupations are up to three times higher than in some professional jobs and mental health, with known indirect links to higher mortality, for those in the poorest quality jobs is as bad as for the unemployed.[5,6] This might reduce the beneficial health effects associated with job gains.

Estimated impact of the overall 2010/11-2021/22 fiscal changes on life expectancy and inequalities in life expectancy.

		Change in life expectancy (weeks)		Change in inequalities			
Scenario	Deaths	All	Most deprived quintile	Absolute gap[1] (weeks)	Relative difference[2] (%)	SII[3] (weeks)	RII[3] (%)
**Females**							
All tax/welfare reforms	+1041	-20	-31	+21	+0·5%	+25	+6%
206,400 people into work	-152	+6	+11	-7	-0·2%	-9	-2%
Total	+889	-14	-20	+14	+0·3%	+16	+4%
**Males**							
All tax/welfare reforms	+1013	-23	-34	+24	+0·7%	+29	+5%
206,400 people into work	-208	+8	+16	-11	-0·3%	-14	-2%
Total	+805	-15	-18	+13	+0·4%	+15	+3%

[1] Absolute gap: Life expectancy in the least deprived areas minus that in the most deprived areas.

[2] Relative difference: Life expectancy in the least deprived areas divided by that in the most deprived areas.

[3] SII, Slope Index of Inequality; RII, Relative Index of Inequality. These are linear regression-based indices that account for absolute and relative health differences, respectively, across the whole socioeconomic gradient.[7]

References

1. Portes J, Reed H. The cumulative impact of tax, social security and public spending decisions in Scotland: Summary report. 2019; .

2. Office for National Statistics. Family Resources Survey, 2014/15. 2019; Available at: https://www.gov.uk/government/uploads/system/uploads/attachment_data/file/531242/family-resources-survey-2014-15.pdf. Accessed Feb 5, 2020.

3. National Records of Scotland. Death records. 2016; Available at: https://www.nrscotland.gov.uk/statistics-and-data/statistics/statistics-by-theme/vital-events/deaths. Accessed Feb 6, 2020.

4. Eurostat. Revision of the European standard population: report of Eurostat's task force. 2013; Available at: https://ec.europa.eu/eurostat/documents/3859598/5926869/KS-RA-13-028-EN.PDF/e713fa79-1add-44e8-b23d-5e8fa09b3f8f. Accessed Feb 6, 2020.

5. Chandola T, Zhang N. Re-employment, job quality, health and allostatic load biomarkers: prospective evidence from the UK Household Longitudinal Study. *Int J Epidemiol* 2018; **47**(1): 47-57.

6. Katikireddi SV, Leyland AH, McKee M, Ralston K, Stuckler D. Patterns of mortality by occupation in the UK, 1991–2011: a comparative analysis of linked census and mortality records. *Lancet Public Health* 2017; **2**(11): e501-12.

7. Pamuk ER. Social class inequality in mortality from 1921 to 1972 in England and Wales. *Popul Stud* 1985; **39**(1): 17-31.
